# The outcomes of surgical treatment via transsphenoidal approach for patients with nonfunctioning pituitary adenoma: a single institution’s experience

**DOI:** 10.1080/07853890.2022.2140449

**Published:** 2022-11-04

**Authors:** Yoon-Hee Choo, Youngbeom Seo, Oh-Lyong Kim

**Affiliations:** a Department of Intensive Care, Seoul National University Hospital, Seoul, Republic of Korea; b Department of Neurosurgery, Yeungnam University Hospital, Yeungnam University College of Medicine, Daegu, Republic of Korea; c Health Insurance Review and Assessment Service, Daegu, Republic of Korea

**Keywords:** Pituitary adenoma, pituitary surgery, transsphenoidal surgery, recurrence, risk factor

## Abstract

**Background:**

Nonfunctioning pituitary adenoma is a primary benign brain neoplasm and the transsphenoidal approach is known for a safe and effective first-line surgical treatment for pituitary tumours. The aim of this study was to retrospectively analyse the outcomes of the transsphenoidal approach for nonfunctioning pituitary adenomas treated at a single institute.

**Methods:**

A total of 181 patients who underwent transsphenoidal approach with nonfunctioning pituitary adenoma at a single institute from March 1998 to November 2018 were included in this study. Ninety-six (53.0%) men and 85 (47.0%) women aged 21–79 years were included. The median outpatient follow-up duration was 58 months, and the median magnetic resonance imaging follow-up duration was 54 months. We assessed the surgical and clinical outcomes, complications, hormonal outcomes and recurrence tendency.

**Results:**

The overall total resection rate of a transsphenoidal approach for nonfunctioning pituitary adenoma was 84.0%. Visual impairment was improved after surgery in 115 (93.5%) of 123 patients. Of the 80 patients who complained of preoperative endocrine dysfunction, 62 (77.5%) patients recovered normal postoperative endocrine function. Diabetes insipidus, which occurred in 22 (12.2%) patients, was the most common complication. A total of 21 (11.6%) patients showed recurrence on average 57.6 months after surgery. The average recurrence period after surgery was 96.3 months in the total resection group of 6 patients and 42.1 months in the subtotal resection group of 15 patients. In multivariate analysis, the extent of resection was identified as a significant predictor of tumour recurrence with a hazard ratio of 6.093 and a *p*-value of 0.002.

**Conclusions:**

It is meaningful to report long-term surgical results within a single institution, and through this, it was reconfirmed that transsphenoidal approach is an effective and safe treatment for nonfunctioning pituitary adenoma. Long-term follow-up is required due to the possibility of recurrence. In addition, performing total resection during surgery helps to lower the risk of recurrence.

**KEY MESSAGES**
- Transsphenoidal approach is an effective and safe treatment modality for pituitary adenoma.
- Complete resection is a significant predictor for the recurrence of pituitary adenoma
- Long-term follow-up is necessary for the treatment of nonfunctioning pituitary adenomas.

## Introduction

Pituitary adenoma accounts for 10–15% of all primary intracranial tumours [1]. Nonfunctioning pituitary adenoma (NFPA) is a primary benign brain neoplasm with a prevalence of 14–54% among all pituitary adenomas [2,3]. The clinical symptoms of NFPAs vary. Some patients are asymptomatic, while others have signs and symptoms such as headache, visual impairment and hypopituitarism due to mass effects on surrounding structures [3,4].

Microadenomas or asymptomatic and relatively small macroadenomas (1–2 cm) can be carefully observed [5]. However, if there is tumour growth or the development of symptoms or anatomical signs of impending visual loss, surgical treatment should be considered [5,6]. The first surgery for a pituitary tumour was performed using a transcranial subtemporal approach in 1892 [7]. Since then, numerous transcranial approaches have been attempted, however, the morbidity and mortality rates are high. Since the first surgical treatment with a transsphenoidal approach (TSA) for pituitary tumour was first attempted by Hermann Schloffer in 1907 [8], a TSA under endoscopy or microscopy has been found to be a safe and effective first-line surgical treatment for pituitary tumours because it is less invasive and has significantly less morbidity [4,9]. As Lee et al. [10] reported a 60% of 10-year progression-free survival rate and Dallapiazza et al. [11] reported 80% and 21% rates of that with gross total resection (GTR) and subtotal resection, respectively, long-term follow-up is necessary for the treatment of NFPAs. The recurrence rate of NFPAs has been reported as 12–66%, depending on treatment conditions [10–13]. Bernat et al. [14] reported that the probability of recurrence at 10 years was 47.2%, respectively.

Herein, we aimed to assess preoperative radiological characteristics, clinical and endocrinological outcomes, surgical outcomes, recurrence rates and factors affecting recurrence for patients with NFPAs who underwent TSA surgery at a single institute.

## Materials and methods

The study was approved by the Institutional Review Board of Yeungnam University Hospital. We retrospectively reviewed 181 patients with clinically and pathologically diagnosed NFPAs who underwent transsphenoidal surgery at Yeungnam University Hospital from March 1998 to November 2018. The patients included in this study were clinically and pathologically diagnosed with NFPA, had a clinical follow-up of at least 6 months, and had at least one postoperative magnetic resonance imaging (MRI) follow-up. Conversely, patients who did not have NFPAs, patients with a clinical follow-up of less than 6 months or who were lost to follow-up, or patients without postoperative MRI were excluded. Among 314 patients who underwent TSA surgery with suspected pituitary adenoma on MRI, the following patients were excluded: 15 patients with a diagnosis other than pituitary adenoma on pathological biopsy, 85 patients with functional pituitary adenomas, 20 patients without postoperative MRI, and 13 patients with a follow-up period of less than 6 months. All Data were obtained from the electronic medical records of Yeungam University Hospital. Patient consent was waived due to study design. Surgical indications were as follows: the patients complained of symptoms, the size of the mass increased, there were anatomical signs of impending visual loss, or the patients wanted to undergo surgery even if there were no symptoms.

We initially diagnosed pituitary adenoma based on MRI and evaluated radiologic characteristics such as size, parasellar extension of the tumour towards the cavernous sinus according to the grading system proposed by Knosp et al. [15], and sellar invasion and suprasellar extension by the Hardy classification proposed by Hardy et al. [16]. We defined cavernous sinus invasion as grades 3 and 4 of the Knosp grading system. Pre- and postoperative physical, neurological, ophthalmological and endocrine function evaluations were performed. In particular, visual impairment was clinically assessed and subsequently confirmed through visual function examination, including visual acuity and visual field defects detected by automatic perimetry performed by ophthalmologists. At the same time, basal serum hormonal concentration assessment was performed to evaluate pituitary function; the parameters of pituitary function included growth hormone (GH), somatostatin-C, prolactin, thyroid-stimulating hormone (TSH), triiodothyronine, thyroxine, free thyroxine, adrenocorticotropic hormone (ACTH), cortisol, follicle-stimulating hormone, luteinizing hormone, and testosterone or oestrogen (for male or female patients, respectively). The hormonal status was classified as follows: normal hormonal function, increased prolactin (stalk effect [17]), GH deficiency, TSH deficiency, gonadotroph deficiency and ACTH deficiency.

### Surgical treatment

At our institution, most patients with pituitary lesions underwent surgical resection with a microscopic TSA surgery until 2010. Since endoscopic TSA surgery equipment was introduced in early 2010 at our institution, endoscopic TSA surgery was performed at the same time as microscopic TSA surgery. The surgery aimed to remove as much of the tumour mass as possible with the aim of complete removal. The sellar floor defect was reconstructed with a multilayered reconstruction technique using fat tissue, autologous bone, absorbable fibrin sealants, and/or a nasoseptal flap [6,18]. In addition, we performed lumbar drainage for any suspected cerebrospinal fluid (CSF) leakage during or after surgery.

### Follow-up and tumour control

In this study, pre- and postoperative tumour control, clinical outcomes, endocrinologic outcomes and complications were evaluated. The extent of tumour resection was evaluated by the intraoperative visual judgment of the surgeon and postoperative MRI. “Gross total resection” was defined as case where there was no gross residual mass as judged by the surgeon intraoperatively and no residual mass on postoperative MRI. “Recurrence” was defined as case where a new mass was observed after total resection or the volume of the remaining mass increased after subtotal resection on follow-up MRI. When recurrence was diagnosed on follow-up MRI, conservative treatment, secondary operation, radiotherapy, or secondary operation and radiotherapy were subsequently performed according to the individual conditions. The clinical outcomes of symptoms were classified into 3 categories: improved, no change, and worsened. With continuous follow-up at the outpatient clinic, “improved” was defined as the subjective improvement of the symptoms or, in particular, for visual impairment, as objective improvement in the postoperative ophthalmic examination. In contrast, “worsened” was defined as the subjective or objective deterioration of symptoms evaluated through a process similar to “improvement”. During the follow-up period, an endocrinologic assessment was performed under additional/adjuvant hormonal replacement by an endocrinologist. The hormonal outcome was classified as “normalized” or “dysfunction” and recategorized as the “normalized”, “1 or 2 dysfunction”, or “3 or more dysfunction” groups.

### Statistical analysis

The results were analysed using IBM SPSS Statistics software (version 20.0 [IBM Corp., Armonk, New York, USA]). Chi-square tests were used to evaluate tumour control outcomes according to cavernous sinus invasion. Linear-by-linear association analysis was used to evaluate tumour control outcomes associated with tumour size, the Hardy classification and Knosp grade. Kaplan–Meier curve analysis was used to evaluate the risk of tumour recurrence according to the extent of tumour resection. Factors predictive of tumour recurrence were entered into the Cox proportional hazards model. A value of *p* ≤ 0.05 was considered statistically significant.

## Results

This study included 181 NFPA patients, with 96 men (53.0%) and 85 women (47.0%). The median age was 54 years (range, 21–79 years). The median clinical follow-up duration was 58 months (range, 6–255 months), and the median MRI follow-up duration was 54 months (range, 1–259 months). Based on preoperative MRI, 5 of 181 patients (2.8%) had microadenomas, which were defined as tumours smaller than 10 mm; 153 patients (84.5%) had macroadenomas, which were 10 mm or larger and smaller than 40 mm; and 23 patients (12.7%) had giant adenomas, which were 40 mm or larger. Based on the Hardy classification [16] and preoperative MRI to evaluate sellar invasion, 5 patients (2.8%) were grade 0, 28 patients (15.5%) were grade 1, 65 patients (35.9%) were grade 2, 43 patients (23.8%) were grade 3, and 40 patients (22.1%) were grade 4. For suprasellar extension with the Hardy classification [16], 26 patients (14.4%) were stage A, 48 patients (26.5%) were stage B, 48 patients (26.5%) were stage C, 25 patients (13.8%) were stage D, and 34 patients (18.8%) were stage E. Regard to the Knosp grade [15], 13 patients (7.2%) were grade 0, 41 patients (22.7%) were grade 1, 65 patients (35.9%) were grade 2, 3 patients (1.7%) were grade 3, and 59 patients (32.6%) were grade 4. Cavernous sinus invasion corresponding to the Knosp grades 3 and 4 was observed in 62 patients (34.3%). The following preoperative hormonal dysfunctions were identified: GH deficiency in 30 patients (16.6%), TSH deficiency in 27 patients (14.9%), gonadotropin deficiency in 34 patients (18.8%), ACTH deficiency in 36 patients (19.9%), and increased prolactin caused by stalk compression [17] in 40 patients (22.1%). TSA surgery was performed in two ways: using a microscope in 164 patients (90.6%) and using an endoscope in 17 patients (9.4%). These characteristics of the 181 patients are summarized in Table 1.

**Table 1.** Characteristics of 181 cases enrolled to study.

Characteristic	Number (%)
Gender (male/female)	96 (53.0) / 85 (47.0)
Median age (years)	54 (21–79)
Median follow-up duration (months)	
Outpatient clinic	58 (6–255)
Magnetic resonance image	54 (1–259)
Surgical approaches	
using microscope	164 (90.6)
using endoscope	17 (9.4)
Tumour size	
Microadenoma (<10 mm)	5 (2.8)
Macrodaenoma (≥10 mm, <40 mm)	153 (84.5)
Giant adenoma (≥40 mm)	23 (12.7)
Hardy classification	
Grade 0	5 (2.8)
Grade 1	28 (15.5)
Grade 2	65 (35.9)
Grade 3	43 (23.8)
Grade 4	40 (22.1)
Stage A	26 (14.4)
Stage B	48 (26.5)
Stage C	48 (26.5)
Stage D	25 (13.8)
Stage E	34 (18.8)
Knosp grade	
Grade 0	13 (7.2)
Grade 1	41 (22.7)
Grade 2	65 (35.9)
Grade 3	3 (1.7)
Grade 4	59 (32.6)
Cavernous sinus invasion (Knops gradeS 3 & 4)	62 (34.3)
Preoperative hormonal dysfunction	
GH deficiency	30 (16.6)
TSH deficiency	27 (14.9)
Gonadotropin deficiency	34 (18.8)
ACTH deficiency	36 (19.9)
Increased prolactin (stalk effect)	40 (22.1)

GH: growth hormone; TSH: thyroid-stimulating hormone; ACTH: adrenocorticotropic hormone.

**Figure 1.**
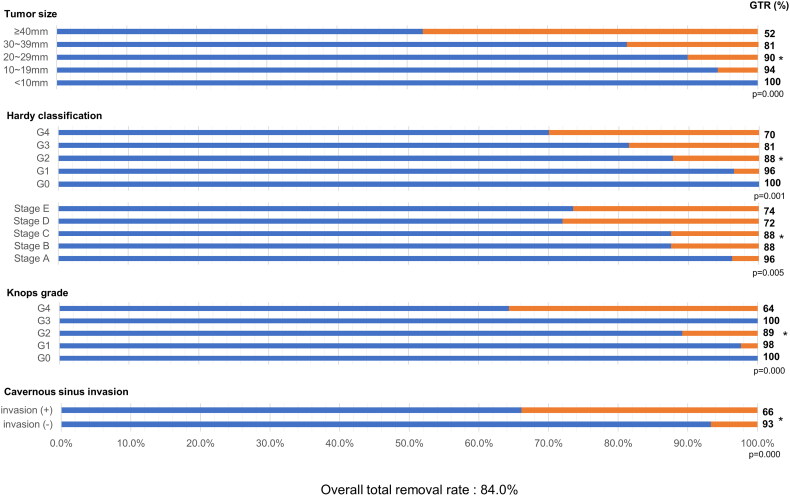
Total resection rate according to each factor of 181 patients enrolled in the study. Linear-by-linear association analysis was used to evaluate surgical outcomes associated with tumour size, the Hardy classification and Knosp grade. Chi-square tests were used to analyse cavernous sinus invasion as prognostic factor. GTR: gross total resection.

The overall total removal rate was 84.0% (152 of 181 patients). As summarized in Figure 1, the GTR rate for each feature of the mass was identified, and the significance of each group and GTR rate was confirmed. The GTR rate according to tumour size was 100% (5 of 5) for tumours smaller than 10 mm, 94.3% (33 of 35) for tumours 10 mm or larger and smaller than 20 mm, 90.0% (63 of 70) for tumours 20 mm or larger and smaller than 30 mm, 81.3% (39 of 48) for tumours 30 mm or larger and smaller than 40 mm, and 52.2% (12 of 23) for tumours 40 mm or larger. As the tumour size increased, the tendency of the GTR rate to decrease was significant (*p* < 0.001). The GTR rate according to the degree of sellar invasion based on the Hardy classification [16] was 100% (5 of 5) for grade 0, 96.4% (27 of 28) for grade 1, 87.7% (57 of 65) for grade 2, 81.4% (35 of 43) for grade 3, and 70.0% (28 of 40) for grade 4; as the sellar invasion became more severe, the GTR rate significantly decreased (*p* = 0.001). The GTR rate according to suprasellar extension based on the Hardy classification [16] was 96.2% (25 of 26) for stage A, 87.5% (42 of 48) for stage B, 87.5% (42 of 48) for stage C, 72.0% (18 of 25) for stage D, and 73.5% (25 of 34) for stage E, which also showed a significant decrease (*p* = 0.005). Furthermore, according to the Knosp grading system [15], as the parasellar extension of the tumour towards the cavernous sinus became more severe, the GTR rate decreased significantly (*p* < 0.001): 100% for grade 0 (13 of 13), 97.6% for grade 1 (40 of 41), 89.2% (58 of 65) for grade 2, 100% (3 of 3) for grade 3, and 64.4% (38 of 59) for grade 4. In addition, the GTR rate was significantly lower for patients with cavernous sinus invasion corresponding to the Knosp grades 3 and 4 than for patients without this feature (*p* < 0.001).

A total of 123 patients complained of visual impairments, including decreased visual acuity, visual field defects, and ophthalmoplegia at the initial presentation. In 115 of 123 patients (93.5%), the visual impairments improved after TSA surgery. Other symptoms, such as headache, dizziness, and loss of consciousness, which are considered mass effects of tumours, improved in 64 of 71 (90.1%), 10 of 11 (90.9%), and 1 of 2 patients (50.0%), respectively. At preoperative assessment, 70 of 181 patients (38.7%) had 1 or 2 hormonal dysfunctions, and 10 of 181 patients (5.5%) had 3 or more hormonal dysfunctions. At the final assessment, 55 of 70 and 7 of 10 patients had recovered and achieved a normal endocrinologic status with 78.6% and 70.0% improvement rates, respectively. The clinical and endocrine functional outcomes of surgically treated NFPAs are summarized in Table 2.

**Table 2.** Clinical and endocrine functional outcomes of patients with surgically treated nonfunctioning pituitary adenoma.

		Initial assessment, *n*	Postoperative change, *n*			Improvement rate, %
			improved	No change	Worsened	
Mass effect	Visual impairment	123	115	8	0	93.5
	Headache	71	64	7	0	90.1
	Dizziness*	11	10	1	0	90.9
	Loss of consciousness	2	1	1	0	50.0
Endocrinologic dysfunction			Normalized	1 or 2 dysfunction	3 or more dysfunction	Normalized rate, %
	Preoperative 1 or 2 endocrinologic dysfunction	70	55	8	7	78.6
	Preoperative 3 or more endocrinologic dysfunction	10	7	1	2	70.0

*6 of 11 patients complained of headache and visual impairment at the same time.

Complications after surgery for NFPAs are detailed in Table 3. CSF leakage occurred in 6 patients (3.3%), diabetes insipidus (DI) occurred in 22 patients (12.2%), panhypopituitarism occurred in 3 patients (1.7%), hyponatremia occurred in 10 patients (5.5%), 1 patient (0.6%) complained of low-pressure headache, meningitis was diagnosed in 1 patient (0.6%), postoperative hematoma occurred in 1 patient (0.6%), and another patient (0.6%) complained of abnormal function of the olfactory nerve.

**Table 3.** Complications of transsphenoidal surgery in this study.

Complication	Number of patients (%)
CSF leakage	6 (3.3)
Diabetes insipidus	22 (12.2)
Panhypopituitarism	3 (1.7)
Hyponatremia	10 (5.5)
Low-pressure headache	1 (0.6)
CNS infection(meningitis)	1 (0.6)
Postoperative hematoma	1 (0.6)
Dysfunction of olfactory nerve	1 (0.6)

CSF: cerebrospinal fluid; CNS: central nervous system.

**Figure 2.**
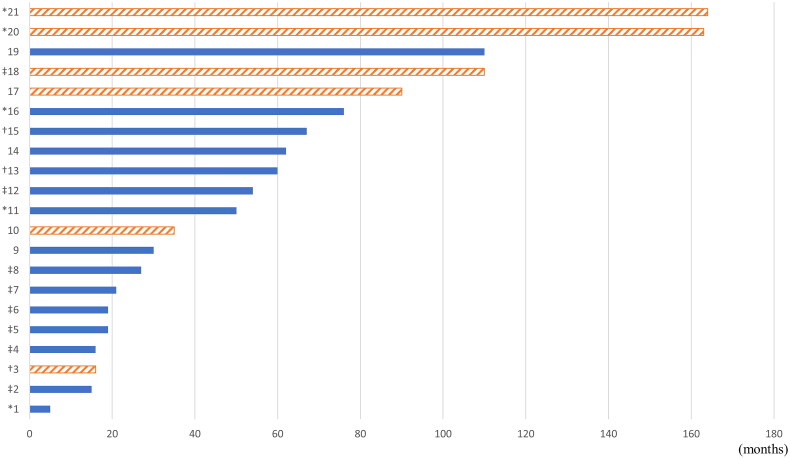
Time interval until recurrence after the first operation and additional treatment for each case of recurrence. The boxes filled with diagonal stripes indicate the cases of recurrence after total resection at the first operation, and the boxes filled with solid colour indicate the cases of recurrence after subtotal resection at the first operation. *Second operation, †Second operation and radiotherapy, ‡Radiotherapy only, no marks; observation or loss to follow-up.

**Figure 3.**
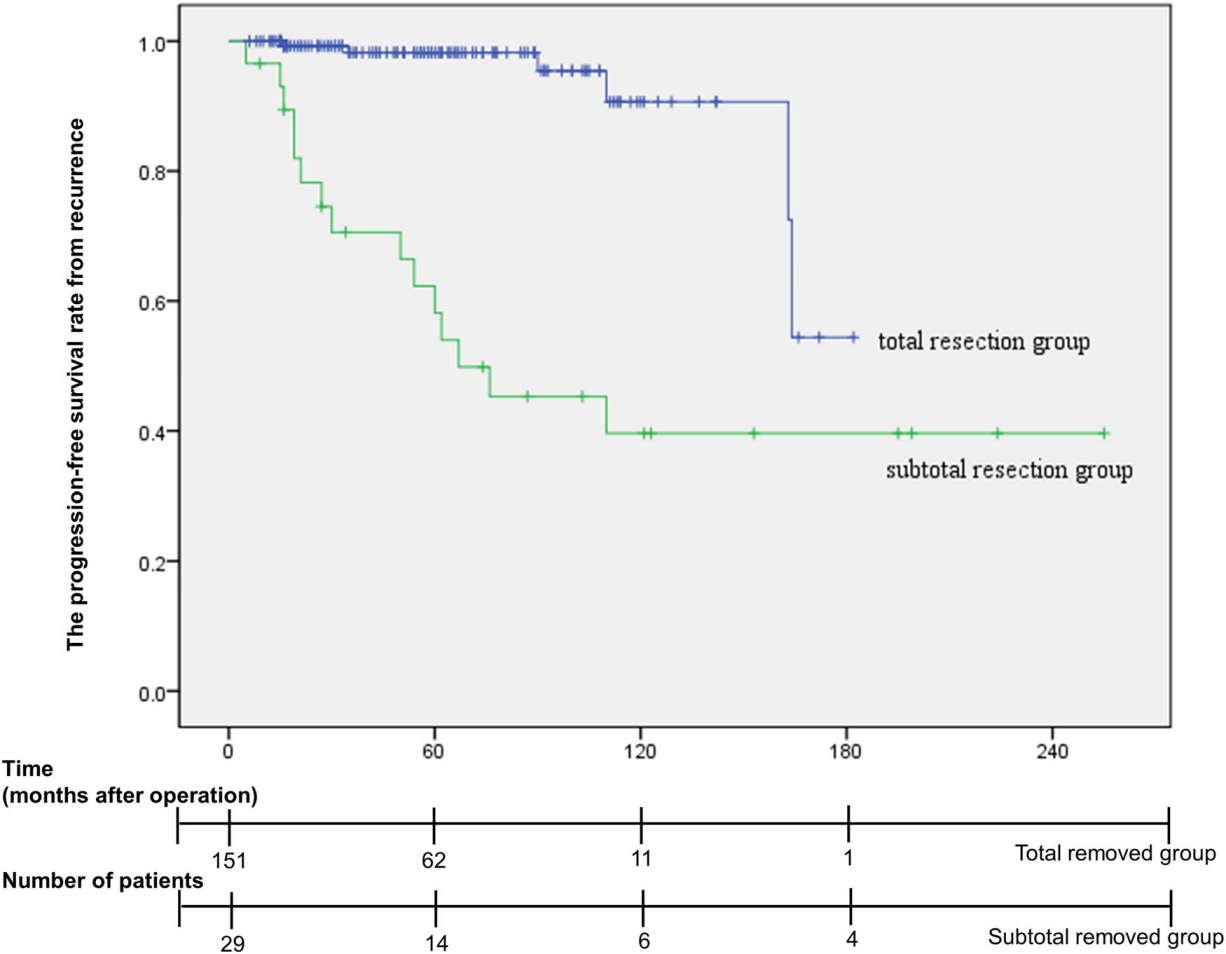
Kaplan–Meier curve of the tumour control rate from recurrence according to the tumour resection extent at the primary operation. In the total resection group at initial operation, the progression-free survival rate was 99.3%, 98.2%, 95.4%, and 54.4% at 19, 55, 118, and 149 months, respectively. In the subtotal resection group at initial operation, the progression-free survival rate was 96.6%, 66.4%, 54.0%, and 39.6% at 5, 13, 62, and 110 months, respectively. There was a significant difference in tumour recurrence between the total and subtotal resection groups (*p* < 0.001).

A total of 21 recurrent cases were reported; 6 cases recurred after total resection, and 15 cases recurred from the remnant mass after subtotal resection. The mean time to recurrence was 57.6 months for all recurrent cases, 96.3 months (range, 16–164 months) for total resection cases, and 42.1 months (range, 5–110 months) for subtotal resection cases. Five patients underwent secondary surgery (TSA for 4 patients and transcranial surgery for 1 patients), 3 patients underwent secondary surgery and radiotherapy, 8 patients underwent only radiotherapy, and 5 patients were treated with conservative treatment. The time interval until recurrence and additional treatment are shown in Figure 2. According to Kaplan–Meier curve analysis, as shown in Figure 3, the progression-free survival rate in the group with total resection at initial operation was 99.3%, 98.2%, 95.4% and 54.4% at 19, 55, 118, and 149 months, respectively. In comparison, in the subtotal resection group, the progression-free survival rate was 96.6%, 66.4%, 54.0%, and 39.6% at 5, 13, 62, and 110 months, respectively. In the univariate analysis, extent of resection, size of tumour, Hardy classification, Knosp grade, cavernous sinus invasion were significantly associated with tumour recurrence. In the multivariate analysis, only extent of resection (hazard ratio: 6.093, *p* = 0.002, 95% confidence interval: 1.936–18.909) was a significant prognostic factor relating tumour recurrence. Statistical analysis was summarized in Table 4. Statistical analyses were shown in Table 4.

**Table 4.** Statistical analysis for prognostic factor relating tumour recurrence.

	Recurrence of tumour				
	Univariate		Multivariate		
	HR	*p*-value	HR	*p*-value	95% CI
Extent of resection	10.446	0.000	6.093	0.002	1.963–18.909
Gender	1.324	0.525	ND		
Age	0.987	0.484	ND		
Size of tumour	1.076	0.000	1.033	0.281	0.974–1.094
Hardy classification for sellar invasion	2.309	0.001	1.293	0.467	0.647–2.581
Hardy classification for suprasellar extension	1.771	0.001	1.045	0.868	0.619–1.764
Knosp grade	1.866	0.002	2.932	0.288	0.403–21.344
Cavernous sinus invasion	3.712	0.000	0.003	0.935	0.000–1.067

HR: hazard ratio; CI: confidence interval; ND: not done.

## Discussion

NFPAs are benign tumours arising from the adenohypophyseal cells [19] characterized by the absence of clinical evidence of hormonal hypersecretion in comparison with functioning pituitary adenoma and the major clinical symptoms are caused by local mass effects [3,4]. According to a summary of many studies by Huang et al. [5], the average proportion of macroadenomas (64.7%) is higher than that of microadenoma (35.3%) in NFPAs. In addition, according to the summary of several studies by Natali et al. [3], the prevalence of macroadenoma among NFPAs is reported as 64.8–100%. In this study, we report a 97.2% rate of macroadenoma among NFPAs. This finding may be due to the fact that symptoms of functioning pituitary adenoma, resulting from hormone excess, appear at the stage of microadenoma, whereas most NFPAs are clinically silent at the stage of microadenoma, and then symptoms often appear as a mass effect with the increase in size. While the rate of tumour invasion into the cavernous sinus is reported to vary from 14.8 to 43.5% [20–22], we report a cavernous sinus invasion rate of 34.3% for NFPAs.

### Surgical outcomes and complications of surgically treated NFPAs

In this study, the overall total resection rate of transsphenoidal surgery for NFPAs was 84.0% and showed a significant positive correlation with tumour size, the Hardy classification, Knosp grade, and cavernous sinus invasion. Compared to the reported total resection rate of 60–81% [20,22–24], the total resection rate of TSA surgery at our institute is relatively high. In addition, regardless of whether endoscopic TSA was introduced at our institute early in 2010 or because of the surgeon’s familiarity and preference, microscopic TSA (90.6%, 164 of 181) was performed more often than endoscopic TSA (9.4%, 17 of 181) for NFPAs at our institute. The total resection rate was 82.3% (135 of 164 patients) for microscopic TSA and 94.1% (16 of 17 patients) for endoscopic TSA. Theoretically, endoscopic TSA, which provides a wider intraoperative view, is likely to lead to a higher GTR rate and lower complication rate [18,25]. Although it is still controversial whether endoscopic TSA or microscopic TSA is better in terms of surgical outcomes and complications [9,20,26–28], several studies [9,18,20,24,26,28] reported that endoscopic TSA is superior to microscopic TSA for NFPAs with a GTR rate for endoscopic TSA of 59–92% and for microscopic TSA of 50–83%. Similarly, in a meta-analysis of 8,257 patients with pituitary adenomas, Almutairi et al. [24] demonstrated that endoscopic TSA resulted in a higher rate of GTR as compared to microscopic TSA for NFPAs in a fixed-effect model (71.0% versus 60.7%, P-interaction <0.01). Recently, the use of an endoscopic TSA has tended to be preferred because it is suitable for obtaining a panoramic view of the operative field, and makes it easy to reach the lateral extended and suprasellar space with endoscopic TSA [9,22,28]. Additionally, at our institute, in recent years, endoscopic TSA has mainly been performed due to the advantages of endoscopy. However, since the total number of endoscopic TSA surgery cases is still relatively small, a re-evaluation of the statistics for endoscopy might be necessary after cases accumulate.

Postoperative complications in this study and their incidence are noted in Table 3. DI has been reported to be the most common complication, with a 3.0–10.23% rate following microscopic and endoscopic TSA for NFPA [6,22,27,29]. In this study, 12.2% (22 of 181) of patients experienced DI. All 22 patients had temporary DI, 18 patients improved their symptoms during the 8-day hospitalization period, and 4 patients were treated with desmopressin intermittently for 2 to 15 months in the outpatient clinic. In addition, intra- and postoperative CSF leakage was observed in 3.3% (6 of 181) of patients. Several studies have reported the incidence of CSF leakage as 0.9–7% [6,9,22,27,29,30]. In order to prevent CSF leakage, the sellar floor was basically reconstructed with multilayered technique. Six patients with CSF leakage had intravenous administration of third-generation cephalosporin and aminoglycoside to prevent central nerve system infection. One of six patients with CSF leakage showed spontaneous improvement with bed rest. Five of 6 patients underwent lumbar drainage, and nonetheless, 4 of them underwent reconstruction surgery due to persistent CSF leakage. Meningitis tends to be more prevalent in microscopic TSA than in endoscopic TSA [9,27]. A meta-analysis performed by Ammirati M. et al. [27] showed a 2.1% incidence or meningitis with microscopic TSA and 1.1% with endoscopic TSA. At our clinic, 0.5% (1 of 213) of patients who underwent microscopic TSA with a 39 mm-sized tumour and cavernous sinus invasion by NFPA developed meningitis. The patients with meningitis had intravenous administration of third-generation cephalosporin and netilmicin, a type of aminoglycoside, for 2 weeks and continued third-cephalosporin with oral administration.

### Clinical and hormonal outcomes of surgically treated NFPAs

The clinical symptoms of NFPAs vary from asymptomatic to mass effects on surrounding structures and hypothalamic/pituitary dysfunction. Headache, with 16–75% incidence, is one of the most common symptoms [4,11,21,31]. A total of 39.2% of patients (71 of 181) in this study complained of headache. As the size of the tumour increases, the stretched diaphragm sella activate the pain fibres in the dura [31]. One other common symptom of NFPA is a variety of forms of visual impairment, such as visual field defects, decreased visual acuity, and ophthalmoplegia; visual impairment related to chiasmal compression or pressure on the abducens or oculomotor nerves in the cavernous sinus. Visual impairment has been reported to have a prevalence rate of 31–60.8% [4,10,11,32]. A total of 68.0% of patients (123 of 181) in this study also complained of visual impairment. Symptoms are improved by decompression by removing the tumour that was compressing the surrounding major structures. Penn et al. [6] reported the improvement rate of headache (80–100%) and visual impairment (56.4–90%) after TSA surgery by summarizing several studies. In this study, postoperative improvement rates for headache and visual impairment were similar to those of other studies, 90.1% (64 of 71 patients) and 93.5% (115 of 123 patients), respectively. Hensley et al. [33] reported a case of pituitary adenoma presenting with dizziness and concluded that dizziness is due to another accompanying symptom, such as headache or blurred vision. In practice, almost half of our patients presenting with dizziness (6 of 11) had headache or visual impairment. However, another half of the patients complained of only dizziness. After the operation, dizziness improved in 90.9% (10 of 11) of patients. As the study was performed retrospectively based on medical records, no other cause of dizziness was identified in the records, and whether dizziness is a symptom associated with other symptoms such as headache or visual impairment should be re-evaluated. The high rate of symptom improvement after surgery suggests that dizziness is the main symptom, possibly due to the lack of medical records for other symptoms. Little et al. [32] and Fatemi et al. [34] reported the incidence of hypopituitarism before treatment with NFPAs as 56.2% and 70%, respectively. In the same vein, 80 of 181 patients (44.2%) in this study were diagnosed with various type of preoperative hormonal dysfunction. Prevalence of GH deficiency, TSH deficiency, central hypogonadism, ACTH deficiency adrenal insufficiency and hyperprolactinemia as stalk effect is reported in 30–100%, 8–81%, 36–96%, 15–62%, and 43–73% respectively [32,34,35]. However, we found the incidence of each hormone deficiency was relatively lower than the overall statistics as Table 1. Several surgical studies reported the improvement rate of hypopituitarism after TSA up to 50% [34–36] and an improvement rate of new or worsened hormonal functions after TSA surgery of 5–58% [34,36]. Compared to this finding, at our institute, among the patients who had initial endocrine dysfunction, 78.8% (63 of 80) of patients had improved endocrine dysfunction, and 15.0% (12 of 80) of patients had new or worsened hormonal functions after TSA.

### Predictors for incomplete tumour resection and recurrence

As the size of the tumour increases and the Knosp grade increases, the risk of an incomplete resection increases. Kim et al. [22] and Mooney et al. [37] also reported that tumour size and cavernous sinus invasion and/or a higher Knosp grade were predictors of incomplete resection. Larger tumours had a higher potential for parasellar or suprasellar extension, thus increasing the risk of incomplete resection [15,16]. Since the degree of parasellar extension and the degree of cavernous sinus invasion or the degree of enclosure around the internal carotid artery increase proportionally [15], the difficulty of total resection is well known due to the risk of internal carotid artery injury. Moreover, the introduction of endoscope equipment improves the limitation of the surgical view of the suprasellar lesion; however, there are still limitations since it is not able to cover all fields of operative view.

Chen et al. [4] reported a recurrence rate of 8.3%, and we found a higher recurrence rate of 11.6%. Several studies [11,13,38] reported residual tumour as a factor influencing recurrence, and Dallapiazza et al. [11] reported that cavernous sinus invasion also affected tumour recurrence. We also finally found that there was a 6.093-fold higher risk for recurrence when there was residual tumour present. However, other factors including cavernous sinus invasion, gender, age, size of tumour, the Hardy and Knosp classification were not significantly affected tumour recurrence. As Dallapiazia et al. [11] and Watts et al. [13] reported that tumour volume was not related to the risk of recurrence, we also found a larger the initial tumour size was not significantly associated with a higher the risk of recurrence.

## Limitations

The limitations of the present study include those inherent to retrospective analyses. Also, one of the limitations of this study is that the volume of study is not quite large. Furthermore, as approximately 90% of the procedures were performed with microscopic TSA surgery, the characteristics of microscopic TSA were reflected rather than those of both endoscopic and microscopic TSA. However, despite these limitations, our study, together with a previously reported series, contributes to knowledge of the safety and effectiveness of surgical treatment for pituitary adenomas.

## Conclusions

TSA surgery is an effective and safe treatment for NFPA with a low rate of mortality. Although the hormonal outcomes were not substantially different from those in previous studies, surgical and clinical outcomes were quite favourable. The presence of residual tumour is an important risk factor for tumour recurrence, as in previous studies, and long-term follow-up is necessary.

## Data Availability

The data are available from the corresponding author upon reasonable request and with permission from the Institutional Review Board of Yeungnam University Hospital.
